# Phenotypic Plasticity Opposes Species Invasions by Altering Fitness Surface

**DOI:** 10.1371/journal.pbio.0040372

**Published:** 2006-10-31

**Authors:** Scott D Peacor, Stefano Allesina, Rick L Riolo, Mercedes Pascual

**Affiliations:** 1 Department of Fisheries and Wildlife, Michigan State University, East Lansing, Michigan, United States of America; 2 Great Lakes Environmental Research Laboratory (National Oceanic and Atmospheric Administration), Ann Arbor, Michigan, United States of America; 3 Department of Ecology and Evolutionary Biology, University of Michigan, Ann Arbor, Michigan, United States of America; 4 Center for the Study of Complex Systems, University of Michigan, Ann Arbor, Michigan, United States of America; McGill University, Canada

## Abstract

Understanding species invasion is a central problem in ecology because invasions of exotic species severely impact ecosystems, and because invasions underlie fundamental ecological processes. However, the influence on invasions of phenotypic plasticity, a key component of many species interactions, is unknown. We present a model in which phenotypic plasticity of a resident species increases its ability to oppose invaders, and plasticity of an invader increases its ability to displace residents. Whereas these effects are expected due to increased fitness associated with phenotypic plasticity, the model additionally reveals a new and unforeseen mechanism by which plasticity affects invasions: phenotypic plasticity increases the steepness of the fitness surface, thereby making invasion more difficult, even by phenotypically plastic invaders. Our results should apply to phenotypically plastic responses to any fluctuating environmental factors including predation risk, and to other factors that affect the fitness surface such as the generalism of predators. We extend the results to competition, and argue that phenotypic plasticity's effect on the fitness surface will destabilize coexistence at local scales, but stabilize coexistence at regional scales. Our study emphasizes the need to incorporate variable interaction strengths due to phenotypic plasticity into invasion biology and ecological theory on competition and coexistence in fragmented landscapes.

## Abstract 2

The ability of an organism to respond adaptively to environmental variation--phenotypic plasticity--can have profound and unexpected effects on species interactions and the probability that a species will invade.

## Introduction

Understanding invasion of habitats by new species is a critical ecological problem due to the enormous impact invasive nonnative species are having on ecosystems worldwide [1–4]. Further, invasion is a key component of many ecological processes such as colonization, ecological succession, and community/metacommunity dynamics [5–7], and understanding the invasion process can provide insight into diverse basic research issues in ecology, evolution, biogeography, and ecosystem ecology [8]. The heart of the problem is to understand which factors cause certain species to successfully invade and exert influence, and to identify community characteristics that make it resistant or prone to invasion [9]. Whereas there will certainly be biological details unique to each invasion, we seek to uncover general properties underlying the invasive process.

The ecological processes that affect an invader's success are the same factors that affect the fitness of a resident species. In particular, both resident and invasive species (or populations) must balance risks of mortality and energy gain in the face of environmental variability, in which the densities of interacting species (e.g., resources, competitors, and predators) and abiotic environmental factors change due to both endogenously and exogenously driven factors [5]. Many species adapt to such variation by evolving phenotypic plasticity that allows individual organisms to respond to a variable environment by modifying traits [10–12]. For example, in two aquatic communities that we study [13,14], predator density varies greatly within and between seasons, and prey exhibit plastic behavioral and morphological responses to this variation in a manner that reduces predation risk. Indeed, prey (animals and plants) from many taxa in disparate systems adaptively modify phenotype (e.g., behavior, life history, and physiology) in response to changes in variable biotic and abiotic factors [10–12] with profound effects on community structure and dynamics [15–17].

Does phenotypic plasticity, i.e., an organisms' flexible and adaptive response, in conjunction with environmental variation, affect the process of invasion? Clearly if phenotypic plasticity leads to increased fitness, we would expect phenotypic plasticity to alter a species' ability to invade an environment and to exclude other invading species if established. Plasticity could enhance invasion by increasing fitness under harsh conditions, favorable environments, or a combination of both [18]. Indeed, a number of studies have reported that invasive plant species are more plastic for traits affecting fitness than are noninvasive exotics [18].

In this study we develop a computational model to investigate the effect of phenotypic plasticity on invasion. The model is simple enough to be general, but complex enough to include the interaction of potentially important processes. In particular the model includes stochastic environmental variability and differences in evolutionary history of resident and invasive species. The behavior of consumer individuals is modeled using an individual-based approach [19,20], including a type of genetic algorithm [21,22]. Consequently, the consumer-species *evolve* an adaptive foraging behavior through selection on traits that dictate the probability of eating (foraging effort), affecting species interactions and therefore system dynamics, which feed back to affect individual behavior (see Methods). Thus our approach models an ecological system as a complex adaptive system [23,24].

**Figure 1:**
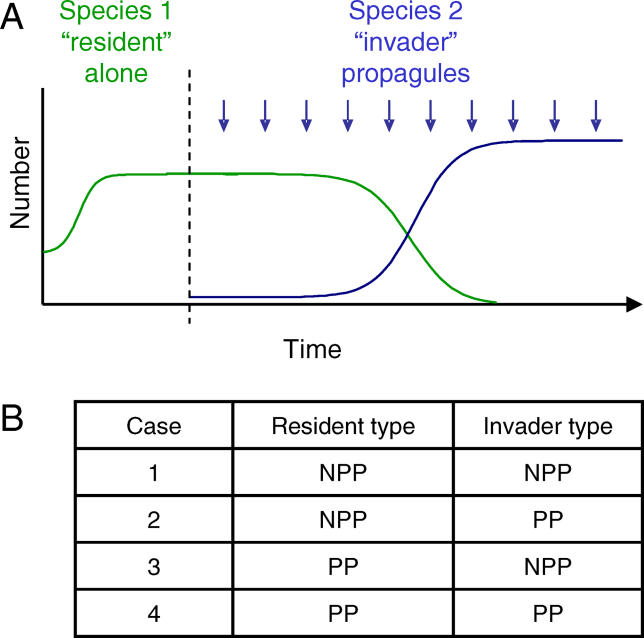
Design of Computational Model (IBM) Experiments (A) Schematic diagram of invasion experiments. Initially, a resident species populates the habitat with a finite initial density and is given a finite time period (dashed vertical line) in isolation sufficiently long to evolve an adaptive behavioral strategy. Thereafter individuals from the “invader” species are added to the habitat. In some cases, the resident species will repel invasion, with the invader species density never increasing. In others, as exemplified in the figure, the invader become established and displaces the resident. (B) Invasion experiments as in (A) were performed for all four combinations of residents and invaders as either NPP-consumers (cannot evolve plasticity) or PP-consumers (can evolve plasticity). Note the resident species evolves an adaptive strategy whether it is an NPP- or PP-resident species, the difference being that, unlike in the PP case, in the NPP case, the resident is constrained to find the behavior that optimizes fitness when that behavior must be the same in both environmental states (predator present and absent).

Our general approach is to investigate invasion of an “invader” consumer into a tri-trophic food chain with a predator, a “resident” consumer, and a resource. As is typical for real organisms [10,25], higher foraging effort in the model provides increased reproductive potential, but incurs increased predation risk when predators are present. The resident species is given an initial finite time period to evolve an adaptive phenotype in the variable environment, after which individuals from the invader species disperse at a small fixed probability into the environment (Methods, Figure 1A). Importantly, as in natural systems, invaders do not have a significant evolutionary history in the new habitat. Therefore, the invader is given an inherent disadvantage in the form of a poorer initial foraging strategy. Invasion success is measured as the time to displace the resident consumer species (Figure 1A). In some cases, the invaders or residents are given an advantage in the form of a lower background death probability, representing, e.g., reduced parasite load in invaders or predation from other predators [26,27].

Predation risk serves as a variable environmental factor by fluctuating between predator presence and absence with different degrees of temporal autocorrelation. The choice of predation risk as an environmental variable factor is motivated by the variable predation risk, and ensuing behavioral phenotypic response, in the natural systems that we study (referenced above). However, other aspects of environmental variability should lead to similar results, as long as the trait under consideration is relevant to plastic responses to such fluctuations. For example, a similar model of competition between plant species that do, and do not, exhibit plastic responses to variability in leaf litter (such as variable hypocotyl length as found in *Impatiens capensis* [28]) should yield results similar to those found here. Because we want to measure the influence of phenotypic plasticity on invasions, we use two types of consumers: (1) phenotypic plastic (PP)-consumers that can discriminate between predator presence and absence, and therefore can evolve different foraging effort in these two environmental states, and (2) non–phenotypic plastic (NPP)-consumers that cannot sense predators, therefore evolving an optimal constant foraging effort used both in predator presence and absence. In order to fully compare the possible situations, we investigate the four possible combinations of the resident and invader species as either PP or NPP (Figure 1B).

Phenotypic plasticity had a profound effect on species invasion. As expected, plastic species invaded a habitat with a non-plastic resident species much more quickly than a non-plastic species invaded a habitat with a plastic resident species. However a novel and unforeseen mechanism that affected invasion emerged, which involved the fitness surface (i.e., fitness plotted as a function of two parameters of interest). When resident species were plastic, plasticity caused a steeper fitness surface that effectively acted as a barrier to invasion, even by invaders that were themselves plastic. These results suggest that phenotypic plasticity can strongly affect invasion biology, and we argue that the fitness surface–based mechanism invoked here has implications for competitive interactions in metacommunities.

## Results

In baseline runs of our model without invasion, both consumer types (PP and NPP) evolved to balance energy gain and predation risk. For PP-consumers, phenotypic plasticity (different behaviors) evolved in all runs; individuals evolved a foraging strategy (i.e., a phenotype defined by the probability of eating in predator presence and absence) to eat nearly 100% of the time in predator absence, but close to 0% in predator presence. In contrast, NPP-consumers evolved an “average” strategy for which the probability of eating was approximately 45% (2% standard deviation over time) in predator presence and absence.

**Figure 2:**
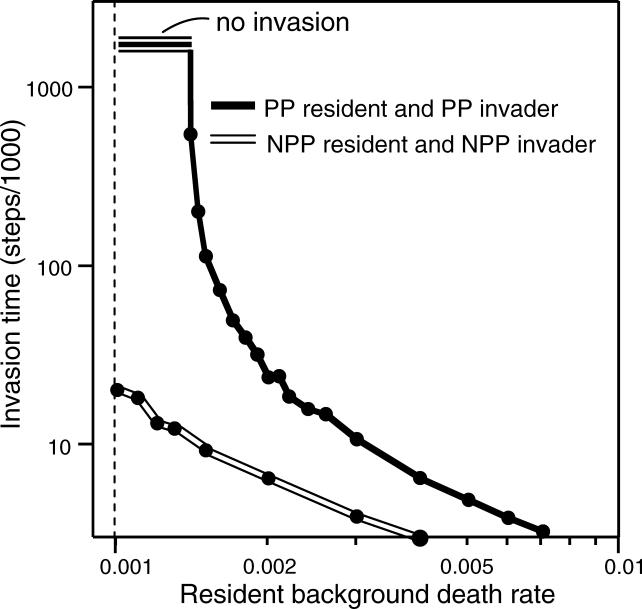
Effect of Plasticity on Invasion Time Mean invasion time (number of steps, log scale) as a function of the background death probability of the resident species, for cases in which the resident and invader both did (PP, solid line) or did not (NPP, empty line) exhibit phenotypic plasticity. The foraging strategy of the individuals from the invader and resident species differed due to different evolutionary histories (Figure 1). Other than this difference, the only difference between individuals from the invader and resident species was their background death probability. Here we show results in which the invader has the default background death probability, equal to 0.001, and that of the resident increases from 0.001 (in which case there is no imposed competitive difference) to 0.01 (in which case the invader has a strong imposed competitive advantage). As expected, the invasion time decreased in both cases (PP and NPP) as the imposed competitive advantage of the invader was increased. Unexpected, however, is that invasion was much slower if both the resident and invader exhibited phenotypic plasticity than if they did not. Note that for a range of background death probability values, from 0.001 to about 0.0015 in which there was an imposed competitive advantage of the invader, there was no invasion with phenotypic plasticity, but there was invasion without phenotypic plasticity.

Phenotypic plasticity had strong effects on invasion time (Figure 2) that were robust to variation in the temporal pattern of predation risk (unpublished data; see Methods). First, consider the two cases in which both invader and resident were either not plastic or plastic (cases 1 and 4 in Figure 1B). When the resident and invader had equal background death probability (i.e., no imposed competitive differences), in the case without plasticity the NPP-invader displaced the NPP-resident species on average in 20,300 steps (standard deviation 6,300 steps, Figure 2). In contrast, there was no invasion (after 2,000,000 steps) in the PP-invader/PP-resident case (Figure 2), and evaluation of dynamics indicate that, in all 20 replicate runs, there was no sign that invasion was ever imminent. Indeed, it was not until the invader was given a large competitive advantage, by increasing the background death probability of the PP-resident to a 40% higher level than the PP-invader, that there were any signs of invasion in the PP-invader/PP-resident case. The difference in invasion time between the PP-invader/PP-resident case and NPP-invader/NPP-resident case continued for larger imposed competitive advantages of the invader, but this difference became smaller at extreme invader-advantage values when invasion time was low in both cases (Figure 2). Including a cost of plasticity did not affect these results (Text S1).

**Figure 3:**
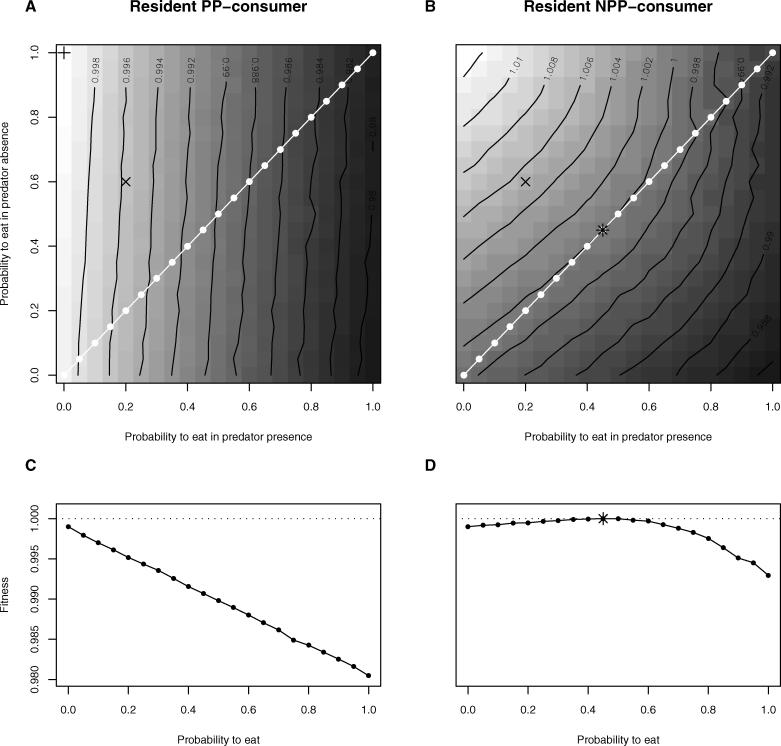
Fitness Surfaces Fitness surfaces for a focal consumer in the presence of either an optimal resident PP-consumer (A) or an optimal resident NPP-consumer (B). Each surface shows fitness as a function of the focal consumer foraging strategy, i.e., of the two traits that describe the probability of eating in predator presence and absence, respectively. Fitness is measured (Methods) in a habitat in which resource levels and dynamics have reached a steady state of the resident species. In (A) the resident PP-consumer has a probability of eating in predator presence and absence of 0.0 and 1.0, respectively (indicated by the plus sign [+]), which is the optimal value for a PP-consumer when alone. In (B) the resident NPP-consumer has a probability of eating of 0.45 in predator presence and absence (indicated by the asterisk [*]), which is the optimal strategy for an NPP-consumer when evolved alone. The fitness of a focal consumer, relative to the respective optimal individual in each figure, is given for each combination of eat probability traits (i.e., in predator presence and absence), where the isopleths in (A) and (B) represent fitness relative to the optimal strategies in the respective cases. For example, an individual with a 0.20 probability of eating in predator presence and 0.60 probability of eating in predator absence (indicated by the x ), had a fitness value of 0.996 with a resident PP-consumer (A) and 1.006 in a habitat with a resident NPP-consumer (B). Because NPP-consumers cannot respond differentially to predation risk, they are restricted to the diagonal (white) lines in (A) and (B), so that the probabilities of eating in predator presence and absence are always equal. Therefore NPP-consumers, when alone, will evolve to the highest point on that line as indicated in (B) by the asterisk (*). Figures (C) and (D) show fitness of the focal consumer as a function of probability to eat when that probability is the same in both predator presence and absence (i.e., along the diagonal in [A] and [B]), in habitats with resident PP-consumers (C) and NPP-consumers (D), respectively.

To understand this pattern, we examined the effect of phenotypic plasticity on the resulting fitness surface (Figure 3). We determined the fitness imparted by various strategies as a function of two traits describing foraging behavior: probability of eating in predator presence and in absence (fitness is measured relative to the optimal strategy; see Methods). As they evolve, PP-consumers gradually move to a peak in the fitness surface, which is the optimal PP-consumer strategy (Figure 3A). In contrast, NPP-consumers were constrained to a single eat-probability value for the two predator states; they therefore evolved to the highest point on the main diagonal of the fitness surface, which is the optimal NPP-consumer strategy (Figure 3B and 3D). In other words, both consumer types evolved to an optimal behavioral strategy corresponding to peaks on the portion of fitness surface available to them. In the case of NPP-consumers, the parameter space available was constrained to a line, and therefore they evolved to a different strategy (point) than did the PP-consumers, because the peak on the entire surface available to the PP-consumers was not on this line (Figure 3A).

**Figure 4:**
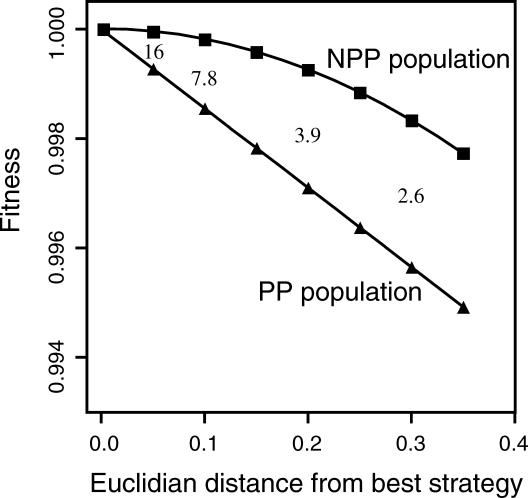
Effect on Fitness of Deviation from Optimal Foraging Strategies Curves show the average fitness of populations that deviate by a fixed Euclidean distance (averaged over a quarter circle for PP-consumers and two points for NPP consumers, see text) from the optimal strategy (i.e., optimal eat probability values), which are at the fitness peaks in Figure 2A (PP-consumers) and Figure 2B (NPP-consumers). Deviations in trait values from the optimum have a markedly larger negative effect on fitness for PP-consumers. Numbers indicate the proportional decrease in fitness for PP-consumers relative to NPP-consumers at different deviations in trait values from the optimal strategy. For example, PP-populations with trait values deviating from the optimum eat probability by 0.1 suffer a fitness reduction 7.8 times greater than NPP-populations with equivalent deviation from the optimum.

Equivalent deviations in the trait value from these optimal strategies had a markedly greater effect on fitness of PP-consumers than NPP-consumers (Figure 4). We can measure the difference in fitness as a function of the Euclidean distance from the evolved strategies (Text S2). For PP-consumers, a constant distance is defined by all points on a quarter circle around the PP optimal strategy, and for NPP-consumers, a constant distance is defined by two points equidistant from the NPP optimal strategy. Moving equivalent distances had much larger negative effects on fitness for PP-consumers (Figure 4). For example, a behavioral strategy that was 0.05 away from the optimal strategy caused, on average, a decrease in fitness of PP-consumers 16 times greater than that caused by an equivalent deviation in fitness of NPP-consumers (Figure 4).

This difference in the effect on fitness of equivalent trait differences, for PP-consumers relative to NPP-consumers, was responsible for the dramatic difference in invasion time. Invading individuals have a phenotype that had not faced selection in the habitat they dispersed to, and therefore were further away from the peak than the resident species, conferring a competitive disadvantage to the invader. The probability of a successful invasion should decrease as a function of this fitness disadvantage. What we find is that, because the landscape was more sharply peaked with phenotypic plasticity, the probability of successful invasion was much lower with, than without, phenotypic plasticity. Therefore the difference in evolutionary history experienced by the resident and invader had a larger negative effect on invasion if consumers (both residents and invaders) were plastic than if they were not.

Consider, for example, the invasion model experiments for which there was no imposed competitive advantage given to the invader (i.e., with equivalent parameter values including background death probability of 0.001 for both resident and invader, Figure 2). There was invasion in the NPP-invader/NPP-resident case, because deviations from the optimal strategy (i.e., that of the resident) only led to a small fitness disadvantage of the invader. Therefore invasion is expected due to stochastic effects between nearly equivalent competitors, and invasion was likely enhanced due to the additional propagule pressure of the invader. In contrast, in the PP-invader/PP-resident case with no imposed competitive advantage, the large fitness disadvantage of the invader conferred by deviating from the optimal strategy prevented invasion (Figure 2). For PP-consumers, the invaders only overcame this “barrier” due to phenotypic plasticity when a large competitive advantage was imposed due to another factor. Here we present results in which this competitive advantage was conferred by manipulating background death probability, but this result was robust to manipulation of other parameters that affect relative competitive ability of the invader and resident.

Next, consider model runs with the NPP-invader/PP-resident and PP-invader/NPP-resident cases (cases 3 and 2, Figure 1B). We found that the NPP-invader was never able to invade a PP-resident (case 3) unless the NPP-invader was given a large competitive advantage (e.g., by giving the PP-resident a much higher background death probability than the NPP-invader). Conversely, a PP-invader was able to very rapidly invade an NPP-resident (case 2), even when they have the same background death probability (i.e., there is no imposed competitive advantage to either species). Relative to the case in which both invader and resident were not plastic, plasticity therefore greatly decreased time-to-invasion if the invader was plastic, but greatly increased time-to-invasion if the resident was plastic. The mechanism underlying this effect of plasticity is distinct from that in the previous cases discussed above. Indeed, these results are to be expected due to the competitive advantage conferred by phenotypic plasticity, which can be seen in the fitness surface (Figure 3A and 3B) in which the fitness of a species with and without plasticity can be compared (i.e., compare fitness of individuals with traits near the PP optimum in the upper left corner to those near the NPP optimum near the middle of the indicated diagonal). The influence of phenotypic plasticity in these cases is, of course, reduced if a cost is associated with plasticity; however, the basic pattern persists even for moderately large associated costs (Text S1).

## Discussion

The results described above show how phenotypic plasticity of a resident species can effectively act as a barrier to invasion. Clearly if phenotypic plasticity increases fitness (including offsetting effects of costs to plasticity), then plasticity will increase an invading species ability to invade [18] or a resident species ability to retard invasions. This expected result was evident by comparing the NPP-resident/NPP-invader case to the NPP-resident/PP-invader or PP-resident/NPP-invader cases. However, our analysis uncovers an additional and unforeseen effect of plasticity when we compare the case in which both invader and resident were plastic to the case in which both resident and invader were not plastic. When both residents and invaders are plastic, an invader individual may be *near* the optimal strategy in regards to trait expression (e.g., probability to eat related to foraging behavior), but because it is not *at* the optimal strategy, it will nevertheless be at a large fitness disadvantage due to the steep fitness landscape induced by phenotypic plasticity, and it will therefore die before reproduction. In contrast, when both residents and invaders are not plastic, an invader that is near the optimal behavioral strategy in regards to trait expression is not at a large fitness disadvantage and is much more likely to gain resources, reproduce, and displace the resident. The effect of phenotypic plasticity therefore has a profound effect on invasion through its effect on the fitness surface.

What is the origin of the large difference in the steepness of the fitness surface with, and without, phenotypic plasticity? Without plasticity, an optimal strategy must balance tradeoffs associated with exhibiting the same phenotype as an environmental factor fluctuates, in our case predator density. Deviations from this strategy will necessarily have opposing effects on overall fitness, which will reduce the net effect. Although similar opposing effects may result from deviations from the evolved behavior with phenotypic plasticity, because strategies are optimized to a particular environmental state (rather than balancing contrasting demands of different states), any deviations will generally have a stronger net negative effect on fitness. For example, in our computational model experiments for PP-consumers, eating with a lower probability in predator absence than the optimal (evolved) strategy will negatively affect fitness with no associated benefit. In contrast, for NPP-consumers, eating less than the optimal strategy will lead to less energy gain, but this negative effect will be opposed by lower predator risk associated with the strategy in predator presence.

The key elements of our model are: (1) competition by the two species for a resource, (2) a variable environment, and (3) a plastic trait that is modified in an adaptive manner in response to this variability. Competition between a resident and an invader is to be expected; environmental variability is ubiquitous; and phenotypic plasticity is widespread in nature. Whereas our investigation uses a variable predation risk and the behavior of consumers in response to this particular aspect of environmental fluctuations, we expect similar results can occur in any other system that includes the above three elements. Further, whereas these arguments suggest generality of the patterns revealed by our individual-based modeling approach, other methods amenable to analysis, such as game theory [29], adaptive dynamics [30,31], and incorporation of phenotypic plasticity into traditional differential equation–based food web models [32,33], may offer promising approaches to address this problem analytically.

There are several approaches that can be taken to empirically evaluate the theoretical prediction proposed here. First, an experimental design analogous to the model design could be used by comparing dynamics of a plastic resident–invader pair in two experimental arenas: one that allows the plastic behavior, and the second that constrains the resident–invader pair to a non-plastic phenotype, thereby mimicking non-plastic species. Methods used effectively previously could be used to simulate predator-induced phenotypic changes and predation [13,34]. One example is provided for a system with zooplankton consumers that respond to predator kairomones (i.e., chemical cues) by migrating deeper at the cost of reduced growth rate due to colder temperatures [35] (Text S3). Second, in systems in which it is not possible to constrain plasticity, it may be possible to combine an empirical examination of plasticity's affect on the fitness landscape with a dynamical model. For example, Stinchcombe and Schmitte [28] (see also [36]) measured fitness of the herbaceous Impatiens capensis as a function of hypocotyl length in bare soil and leaf litter. Using these empirically derived data, they constructed a fitness landscape for I. capensis that experiences variability in leaf litter as a function of encounter frequencies of the two environmental states. This type of derivation could be combined with dynamical models to examine the effect of plasticity on invasion success. Third, invasion properties of species with plastic and fixed (i.e., non-plastic) responses to environmental variation could be compared experimentally. For example, in zooplankton prey communities, some species respond to predator presence through vertical migration, whereas others do not. Lastly, an analysis of invasions by exotic species, in which systems with plastic and non-plastic invader–resident pairs are compared, could yield insight into this prediction. Note that this latter method is distinct from comparing the degree of plasticity of invaders to that of residents or noninvasive exotics [18].

Our theoretical results have clear extensions to the stability of species interactions in general, and consequently to species coexistence and biodiversity. The increase in steepness of the fitness surface caused by phenotypic plasticity will have a destabilizing effect on competitor coexistence at a local scale, because small differences in traits will have much larger effects on fitness. Indeed, this predicted effect on competition is evident in the invasion simulations in which coexistence periods were more pronounced with NPP-invader/NPP-resident pairs. When invasion in the PP-invader/PP-resident case occurred, there was an initial period of low invader density, maintained by pressure from introduced individuals, followed by a fast increase in density and exclusion of the resident. In contrast, in cases of similar invasion time with NPP-invader/NPP-residents, density initially increased steadily followed by long periods of intermediate densities of both the invader and resident. Thus, due to fitness landscape steepness differences, coexistence was much less stable with plasticity than without plasticity. Conversely, the model results predict that phenotypic plasticity will have a stabilizing effect at a larger regional scale (i.e., heterogeneous habitats), because phenotypic plasticity of species that respond adaptively to local variable environmental factors will increase resistance to invasion from the larger regional pool. Our results on invasions, and extensions to competition, thus have implications for the relationship between regional and local patterns of species distributions, e.g., metacommunities [6,37].

Our results exemplify how using an individual-based computational approach [19,20] can elucidate how individual-level behavior generates macroscopic patterns at the community level. Although very simple in representation, the state dependence of an individual's behavioral strategy had a profound effect on invasion at the community level. Importantly, the stabilizing mechanism involving the fitness surface was unforeseen and discovered as we were exploring the parameter spaces [38]. Additional research now can be carried out to examine the generality of the mechanism, and whether and how the mechanism extends to higher-dimensional systems, thus influencing patterns of food web structure and biodiversity.

Phenotypic plasticity and evolution operate on different time scales (plasticity within generations and evolution across generations) but are fundamentally similar in the manner that traits change adaptively to environmental change. Therefore some study results based on evolutionary changes can be extended to predict effects of phenotypic plasticity. Evolution of traits is predicted to reduce competition and increase coexistence of competitors [33,39,40]. Generalizing this result to the effect of phenotypic plasticity suggests that phenotypic plasticity could increase the probability of invasion by reorganizing the food web in a way that increases likelihood of survival of a new species. The process responsible for this predicted effect of plasticity is distinct from that involving the fitness landscape steepness examined in the present study.

The search for ecosystem properties that affect invasions is an intensively studied discipline. For example, environmental variability has been argued to increase species invasions because it can provide footholds, or extend niche space [41,42]. Further, biodiversity is hypothesized to impede invasions by reducing the niche widths or resource levels available to invaders [43–45]. Our study is distinct from these studies by considering the invasion process over longer time periods and adaptation to many cycles of environmental change.

In summary, our results suggest that phenotypically plastic responses to a dynamic environment can strongly impact invasion, coexistence, and therefore biodiversity. Factors that affect species invasions and coexistence are fundamental to ecological communities [46–49], in part because such factors underlie the extraordinary biodiversity of natural systems, and have implications for management of ecosystems. Our study provides an additional mechanism based on adaptive responses to environmental variability that can potentially affect species interactions and invasion.

## Materials and Methods

### Overview of individual-based model.

All computational experiments were carried out with DOVE (Digital Organisms in a Virtual Ecosystem [50]), a computational system for implementing individual-based models (also called agent-based models) with discrete time and space. Each simulation included one habitat (a torus-like grid) populated by plants (which grew logistically to a user-defined per-cell carrying capacity) and a predator species. Cells also may be inhabited by consumers, the focal species of the experiments. In short, a simulation proceeds as follows. First, an initial population of consumers is placed randomly in the habitat, then each time step: (1) the predation risk level is set; (2) plants grow; (3) each consumer uses its behavioral strategy to choose one of two possible actions, eat (and gain biomass) or remain inactive (with no biomass lost or gained); (4) consumers that have accumulated sufficient biomass and have not recently reproduced “mate” and reproduce; and (5) consumers may die due to predation or a random background death process (representing causes external to the modeled food web). These five actions were repeated every time step in the order listed (2 and 3 were interleaved) until the end of a simulation, and measures of interest were recorded (e.g., the extinction time of consumers). Runs were repeated with different random number generator seeds, and results were statistically summarized as described below.

We use fluctuating predation risk to represent variation in an environmental factor in general. Fluctuating predation risk for consumers was generated with a general model that has been used extensively in the literature for discrete states and produces a specified degree of temporal autocorrelation in environmental variability [51]. Specifically, we modeled fluctuating predation risk as a stochastic process using discrete-state Markov chains in which the probability distribution at time *t* + 1 depends only on the state at t according to the equations

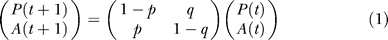


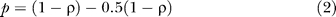


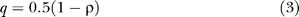
where *P* and *A* are the probability of predator presence and absence, respectively, p and q determine transition probabilities, and ρ is the autocorrelation between the state at times *t* and *t* + 1 ([51] pp 377–380). The autocorrelation function decays exponentially with time. We present results for ρ = 0.8, for which the mean predator presence/absence period was ten time steps, with standard deviation of 9.5 time steps. However, extensive runs over a large range of correlation coefficients (from ρ = 0.95 to −1) and therefore over a large range of mean and variance in predator presence/absence time, indicate that our results are robust to different predator sequences. Results are also robust to a more periodic predator presence/absence sequence, in which a stochastic sequence of predator presence and absence periods with a mean length of ten steps (variance 10) was used.

Our choice of an exogenously driven stochastic environmental variation, which decouples the feedback between prey and predator density, was motivated by observation of predator–prey dynamics in a pond ecosystem. In particular, the tight coupling between predator and prey densities invoked in the majority of dynamic models of multiple species is often unrealistic, because in natural systems the predator and prey dynamics are strongly affected by other species (e.g., predators, competitors, and resources) and by stochastic abiotic factors [52–54]. Further, even in systems with strong or partial coupling, additional mechanisms independent of feedback of predator density on prey dynamics may be operating. The general framework here is one with two competing consumers and environmental variability. The predator itself is specifically introduced to consider environmental variability that is relevant to the plastic trait under consideration.

Each consumer uses its behavioral strategy to select one of two actions, eat or remain inactive, at each time step. To capture the predation risk/energy gain trade-off associated with foraging, the eat action leads to biomass gain and predation risk, whereas inactivity leads to neither. If an animal eats, it remains in the same cell or moves to one of the eight adjacent cells chosen at random and eats 20% of the plant biomass in that cell (computed after subtracting a refuge fraction, 20% of the carrying capacity, that consumers cannot utilize). The eat action also results in an 0.05 probability that the individual will die as a result of predation if the predator is present that time step (there is no predator satiation), whereas there is no predation when a consumer remains inactive or if the predator is absent.

To represent phenotypic plasticity, PP-consumers differed from NPP-consumers by their ability to behave differently in predator presence and absence. The choice of using state-dependent changes in behavior to represent phenotypic plasticity is motivated by our own experimental studies [13,14]. However, results should be general to plasticity in other traits, such as plasticity in morphology, life history, or physiology [10,11,15–17,55]. A plastic behavioral response differs from transient behavioral responses such as fleeing a predator. Rather, with behavioral plasticity, consumers perceive predator presence in the habitat and adopt persistent behavioral modifications (relative to predator absence) whether or not predators are in the immediate proximity [10].

**Figure 5:**
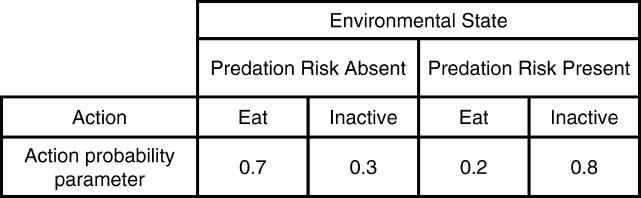
Action Probability Parameters that Determine the Behavioral Strategy for a Single Individual PP-Consumer There are two possible actions for each environmental state (i.e., different predation risks). In this example, the consumer has a 70% probability of performing the eat action (which can include moving to a neighboring cell) if the predator is absent, but 20% if the predator is present. Note that animals from the same species would have the same set of action probability parameters that determine the behavioral strategy, but the probability magnitudes may differ. Behavioral strategies with higher fitness will be selected and therefore “evolve” over time.

PP-consumer behavioral strategies were represented as simple tables that map from each possible discrete environmental state (i.e., predator presence or absence, which represents high or low risk, respectively), to a list of action probability values that specify the probability of eating or remaining inactive. Figure 5 shows one such mapping defining a particular behavioral strategy. In this example, if the predator is absent, the probabilities that the animal will eat or remain inactive are 0.7 and 0.3, respectively, but if the predator is present, those probabilities are 0.2 and 0.8. (Of course the probability values to eat and remain inactive in the same state must add to 1.0, since these define the probability distribution over all possible actions.) NPP-consumers, in contrast, cannot sense predator presence or absence, so their behavioral strategy contains just two action probability values; probability of eating and probability of remaining inactive for all levels of predation risk. In any case, a given PP- or NPP-consumer will choose an action at a given time step by making a draw from the action probability values it has for the level of predation risk at that time, and execute whatever action it happens to pick, perhaps accumulating additional biomass and risking predation, as described above.

When a consumer's accumulated biomass exceeds a threshold, and if it has not reproduced in the past 20 time steps, it becomes “fertile” and then can mate with another fertile conspecific, chosen at random. (The limit of one reproduction per 20 steps gives rise to limited returns on resource acquisition at high eat-probabilities.) When two individuals reproduce, a single offspring receives action probability values from one or both of the “parents” using a standard genetic algorithm [21,22]. That is, the list of action probability values that constitute a consumer's behavioral strategy (e.g., as shown in Figure 5) is treated as a “genome” and each value in the list is considered a “gene” (using the genetic algorithm parlance). An offspring's behavioral strategy is created through sexual reproduction with single-point crossover (including one edge) of the parents' genomes, possibly followed by mutations (with a 0.02 probability per gene of adding Gaussian (μ = 0, σ^2^ = 0.25) noise to the value being mutated). After reproduction, the offspring's action probabilities are re-normalized if needed, each parent's biomass and that of the offspring is set to zero, and the offspring is placed at random in the habitat.

### Invasion experiments.

The habitat initially was populated with a resident consumer species composed of 5,000 individuals with randomized action probability parameters, placed in randomly selected cells. This resident species was allowed to evolve for 100,000 steps, which was sufficient for an optimal (and unchanging) strategy to be achieved. Due to mutations during reproduction, a certain amount of variation always remains in the population. After this time period, a second consumer species, the “invader” species, was allowed to disperse in the habitat: at each time step, a certain number (mean : 0.1 individuals, σ^2^: 0.1, using a binomial distribution B(n,p), n = 1,000, p = 10^−4^) of animals were put into the habitat. The magnitude of this propagule pressure affected invasion time, but not the qualitative nature of the results. The action probability parameters of the invaders were initialized at random, which reflects their lack of evolutionary history in the habitat. For 20 replicated simulations initiated with different random number generator seeds, either the resident excluded the invader for 2,000,000 time steps, or we measured the time it took the invader to exclude the resident (i.e., the resident went extinct).

Experiments were performed for all four combinations of PP-consumers and NPP-consumers as the invader or resident species. Both phenotypic plasticity and the difference in evolutionary history of the resident and invader are predicted to affect invasion. In order to explore the invasion further, we manipulated relative competitive ability of the resident and invader by examining invasion over a range of background death probability of both species. We increased the background death probability of the invader (or resident), in order to increase (or decrease) the relative competitive ability of the resident. Thus, for example, if there was no invasion in cases when all constant parameters of the species were equal (including background death probability), it was possible to increase the background death probability of the resident species and determine the imposed competitive advantage the invader needed for invasion (Figure 2).

Additional model experiments established that (1) resident consumer species extinctions were due to competition from invaders, since species never went extinct when run without invaders for an equal number of time steps with the same parameters as used in the invasion experiments, (2) the evolved behavior strategy was globally stable, i.e., it is an evolutionary stable strategy, (ESS [29]), if we ignore the never-eat strategy that leads to extinction, (3) the number of action probability parameters, which was larger for PP-consumers than for NPP-consumers, did not affect the results (Text S4), and (4) including a cost of plasticity [56–58] did not affect the qualitative nature of the results (Text S1).

We also performed invasion experiments in which the consumers did not evolve in order to verify that unforeseen factors resulting from the evolutionary processes included in the invasion experiments were not responsible for observed patterns (Text S5). In this case the resident species was given the optimal action probability parameters and the invading species was given action probability parameters that were a fixed distance in strategy space from the optimum.

### Fitness surfaces.

We constructed a fitness surface in order to compare the fitness conferred by different behavioral strategies relative to the optimal behavioral strategy (i.e., the one leading to the highest possible fitness). It is important to take into consideration that consumer numbers and density will affect resource levels and dynamics. Therefore the difference in fitness between any two individuals with particular behavioral strategies, which is affected by resource levels and dynamics, will be a function of the density of other individuals in the system, i.e., there is frequency dependence. The fitness surface therefore differed when PP-consumers or NPP-consumers were resident, and it was necessary to examine effects of phenotypic difference on fitness in each scenario separately. Thus, we examined the fitness of a systematic sample of all possible foraging strategies, defined by particular combinations of action probability parameters, relative to the PP-consumer when the PP-consumer is a resident species at steady state, and relative to the NPP-consumer when the NPP-consumer is a resident species at steady state.

We determined the fitness of different “test” species with behavioral strategies, C^T^, relative to the behavioral strategy of the resident species, C^R^, by measuring the population growth rate of the test species in an environment in which the resident species is at steady state (and hence intrinsic population growth rate is equal to 1). By varying the value of the action probability parameters of C^T^ that determine its probability of eating, over all possible combination from 0 to 1 (step 0.05), we could then establish the fitness surface. Unlike in the invasion experiments (described above), the action probability parameters that determine actions for C^R^ and C^T^ were fixed and identical for all individuals within the resident and test populations. Both C^T^ and C^R^ ate, reproduced, and died from predators and random death, as in the invasion experiments.

Our goal was to measure the fitness of individuals with the strategy of the test population, C^T^, in a habitat in which resource dynamics are dictated by C^R^; that is, we wanted to measure the fitness of a species with a particular phenotype (i.e., that of C^T^) in an environment in which C^R^ is resident and at steady state. Therefore we needed to insure that C^T^ does not itself affect resource density and dynamics. One approach would be to use low densities of C^T^. However, using low densities introduces confounding factors that would not allow us to measure the C^T^ fitness (growth rate) precisely. These factors arise from the discrete nature of computational system (i.e., discrete number of individuals and discrete time) combined with the threshold energy needed for reproduction, and from the Allee effect (i.e., at low density some individuals may be ready for reproduction in the absence of a second individual to reproduce with). Both of these factors could underestimate population growth rate. We therefore implemented an alternative method that completely removed the effect of C^T^ on the resources while at the same time overcoming confounding factors associated with low density. We did this by modifying the eat action of C^T^, such that when it ate, it acquired the appropriate amount of resource, but the resource was left unaffected (i.e., the resource contributed to C^T^ growth without actually being removed). Thus, C^T^ growth rate was still affected by all the same factors in our other model runs (i.e., mortality due to predation and background death probability, and growth due to consumption of resources), but it does not affect the system dynamics. This method allowed us to measure the population growth rate of C^T^ in a system in which resource level and dynamics are dictated by C^R^ at steady state. In order to increase the accuracy of our estimate of the population growth rate of C^T^, population growth rate was measured for each C^T^ multiple times and averaged. Resultant fitness surfaces are illustrated in Figure 3. We performed tests of this method using particular cases that have known fitness (such as C^T^ with a strategy the same as C^R^, or C^T^ only affected by background death probability) that confirmed its validity.

## Supporting Information

Text S1 Influence of Cost of Plasticity (34 KB DOC)

Text S2 Quantifying the Effect on Fitness of Deviations from Optimal Behavioral Strategy (33 KB DOC)

Text S3 Example Experimental Design (28 KB DOC)

Text S4 Effect of the Larger Number of Action Probability Parameters for PP-Consumers Than for NPP-Consumers (29 KB DOC)

Text S5 Invasion Experiments without Evolution (29 KB DOC)

## Abbreviations

NPP: non–phenotypic plastic
PP: phenotypic plastic
