# Supplementary material for: Phenotypic Plasticity Opposes Species Invasions by Altering Fitness Surface
Source: PLoS Biol. 2006 Oct 31;4(11):e372. doi: 10.1371/journal.pbio.0040372 (PMC1629039; doi:10.1371/journal.pbio.0040372)
Supplement: Supplementary file 1 [file pbio.0040372.sd001.doc]

**Text S1. Influence of Cost of Plasticity.**

We examine whether costs to plasticity affect the theoretical predictions derived here. We implemented a cost to plasticity by reducing the conversion efficiency of resources into consumer biomass. Our principal interest is to examine whether this cost will affect invasion rates for plastic invader-resident pairs, and thereby modify the invasion time relative to nonplastic invader-resident pairs. Results including a reduction in conversion efficiency ranging from 50% to 80% (i.e. below that used in the base experiments) indicated that a cost did not significantly affect invasion time (Figure S1). Although this testing is necessary to ensure that cost did not affect our conclusions, this result is expected since the cost will not affect the mechanism responsible for the reduced invasion time with plasticity; the cost will act to shift the position of the fitness surface vertically, but will not affect the peak steepness.

For completeness we also examined the effect of cost on the cases with NPP-invader/PP resident pairs, and PP-invader/NPP resident pairs. Clearly we would predict that a cost of plasticity will affect the invasion time in these cases, since it will reduce (or even reverse if cost is very large) the positive effect of plasticity on relative fitness. We explored this problem in a system for which resident and invaders had equivalent background death rates (recall that background death probability was varied in the main text to give the resident or invader an intrinsic competitive advantage). We found that, as predicted, cost of plasticity affected invasion rates. Without a cost, NPP-invaders could not invade a system with PP-residents (Figure 2 in main text), but with a cost of plasticity created by a reduction in conversion efficiency (by at least 92%) invasion by NPP invaders into a system with PP residents took place. Also, without a cost, PP-invaders could invade a system with NPP-residents, but a cost of plasticity by a reduction in conversion efficiency (by at least 85%) stopped invasion. Developing a more comprehensive quantitative analysis of this effect is beyond the scope of this study, though these results show that the computational system produces predicted results, and we believe it likely offers a powerful approach.

**Figure S1. Effect of Cost of Plasticity on Invasion Time.**

Invasion time (mean ± standard deviation) is shown for the PP-invader / PP-resident case in which conversion efficiency was reduced (representing the cost of plasticity) by 80% and 50% for both species for cases in which resident background death probability (bgdp) was equal to 0.0015 and 0.002, and invader bgdp was equal to 0.001 (the default value). For comparison, the invasion time with no cost (0 % reduction), and for the NPP-invader / NPP-resident case are illustrated (note that these points are also shown in Figure 2).
