# Supplementary material for: Phenotypic Plasticity Opposes Species Invasions by Altering Fitness Surface
Source: PLoS Biol. 2006 Oct 31;4(11):e372. doi: 10.1371/journal.pbio.0040372 (PMC1629039; doi:10.1371/journal.pbio.0040372)
Supplement: Supplementary file 2 [file pbio.0040372.sd002.doc]

**Text S2. Quantifying the effect on fitness of deviations from optimal behavioral strategy.**

We compared the effect on fitness of deviations from the action probability parameter values that yielded the optimal strategy. To do this, we (1) found polynomial equations that provided best fits to the fitness surface for the PP-consumers (Figure 3a) and the fitness line for NPP consumers (Figure 3d). The Adjusted R-squared was 0.9999 and 0.9944 for the best fits for the PP and NPP cases, respectively. We then computed the fitness for all combinations of action probability parameters that deviated from the optimum value of PP-consumers (integrating over a quarter circle) and NPP-consumers (averaging two values equidistant from the optimal value).

For PP-consumers, we fit the PP landscape with a quadratic function:

$Fitness(x,y)=ay+bx+cy^{2}+dx^{2}+e$,

where *x* is the probability of eating in predator presence and *y* is the probability of eating in predator absence. The intercept *e* was set equal to (1-death background) = 0.999. We then computed the average value of *Fitness(x,y)* for all values a defined distance, *r*, from the optimal (maximum fitness) strategy *(x=0, y=1)*.

Therefore we intersected the best fit surface with:

$r=\sqrt{x^{2}+\Psi^{2}}$ ,

where *ψ = y-1*. We obtain the average fitness by substituting *r* and *ψ* for *x* and *y* in the fitness equation, integrating the quarter circle from *ψ =-r* to 0, and dividing by *r*:

$$\begin{matrix} Avg(Fitness(r))_{PP}=\frac{1}{r}\int_{-r}^{0} Fitness(\psi,r)d\psi\\ =\frac{1}{r}\int_{-r}^{0} \left(b\sqrt{r^{2}-\psi^{2}}+(c-d)\psi^{2}+(a+2c)\psi+dr^{2}+a+c+e\right)d\psi\\ =\frac{1}{r}\left(\frac{b\pi r^{2}}{4}+\frac{(c-d)r^{3}}{3}+\frac{(a+2c)r^{2}}{2}+r(a+c+e+dr^{2})\right) \\ =\frac{b\pi r}{4}+\frac{(c+2d)r^{2}}{3}+\frac{(a+2c)r}{2}+a+c+e \end{matrix}$$

For NPP-consumers, we fit the NPP “landscape” (the line in which the probability to eat in predator presence and absence was equal, main text Figure3d) with the cubic function

$Fitness(x)=fx^{2}+gx^{3}+e$,

where *x* is the probability of eating (in both predator present and absent states). The intercept *e* was set equal to (1-death background) = 0.999. To compute the effect of a deviation, *r*, from the action probability parameter value at the maximum of the best fit line (probability to eat = 0.453) we averaged the fitness for gene values 0.453*+r* and 0.453*–r*:

$$Avg(Fitness(r))_{NoPP}=\frac{f\left(0.453+r\right)^{2}+g\left(0.453+r\right)^{3}+e+f\left(0.453-r\right)^{2}+g\left(0.453-r\right)^{3}+e}{2}$$

We can now compare *Avg(Fitness(r))*_PP_ and *Avg(Fitness(r))_NPP_* for any given deviation *r*, from the optimal strategy. In order to facilitate comparison we normalized the maximum fitness to 1 (because the fitting was precise only to the third decimal digit).
