# Supplementary material for: Phenotypic Plasticity Opposes Species Invasions by Altering Fitness Surface
Source: PLoS Biol. 2006 Oct 31;4(11):e372. doi: 10.1371/journal.pbio.0040372 (PMC1629039; doi:10.1371/journal.pbio.0040372)
Supplement: Supplementary file 3 [file pbio.0040372.sd003.doc]

**Text S3. Example Experimental Design.**

Here we provide an example experimental design that is analogous to the model design in the main text. The goal is to compare invasion dynamics of a plastic resident-invader pair in two experimental arenas: one that allows the plastic behavior, and the second that constrains the resident-invader pair to a non-plastic phenotype, thereby mimicking non-plastic species. Consider, a system with zooplankton consumers that respond to predator kairomones (i.e., chemical cues) by migrating deeper into the water column [34]. This migration reduces predation risk, but comes at the cost of reduced growth rate due to colder temperatures. Using experimental columns [34], variable predator presence could be created with the method of simulating nonlethal and lethal effects that has been used effectively previously [13,35]; predator kairomones could be dripped into the columns [14,34] to elicit phenotypic responses, and prey could be manually removed to simulate predation (mortality) [13,35]. Two *Daphnia* species (or two clones) that respond to predator kairomones could serve as invader and resident consumers with similar, but not identical, traits. The experiment design for invasions would be conducted in much the same way as our model analysis. The PP-consumer treatment would be established by allowing *Daphnia* to migrate freely in the water columns. The NPP-consumer treatment would be created by constraining the *Daphnia* to a limited range of the water column with screening. Clearly details would vary greatly between systems. However, we believe that the key manipulation of constraining a phenotypic plastic species to perform in a way similar to a nonplastic response could be effective in many plant and animal systems.
