# Supplementary material for: Phenotypic Plasticity Opposes Species Invasions by Altering Fitness Surface
Source: PLoS Biol. 2006 Oct 31;4(11):e372. doi: 10.1371/journal.pbio.0040372 (PMC1629039; doi:10.1371/journal.pbio.0040372)
Supplement: Supplementary file 4 [file pbio.0040372.sd004.doc]

**Text S4. Effect of the Larger Number of Action Probability Parameters for PP-consumers than for NPP-consumers.**

The behavioral strategy of a resident species is dictated by action probability parameters that have been subject to evolution (and so are close to optimal), whereas invading species will have action probability parameters with random values. The probability that an invading individual’s action probability parameters will be close to a high fitness peak combination will decrease with the number of action probability parameters. Thus, we would expect that, even in the absence of the mechanism based on the fitness surface steepness invoked in the main text, invasion in the PP-invader/PP-resident case would be lower than in the NPP-invader/NPP-residents because there are more action probability parameters in PP-consumers than in NPP-consumers. We therefore performed experiments to ensure that the higher action probability parameter number (2 fold given separate genes for predator presence and absence) for PP-consumers was not responsible for the observed difference in invasion rates.

First, with the same parameters used in the simulations described in the main text, we created a duplicate set of action probability parameters (“genes”) in the NPP case, and behavior was dictated by choosing randomly from the two genes sets. Thus there were an equal number of action probability parameters in the NPP and PP case. This modification did not affect the invasion patterns described in the main text. A potential problem arose with this first approach because the values that evolved for the paired action probability parameters sets were negatively correlated, and thus not independent. We therefore used a second approach in which we modified other parameter values so that optimum strategy in the NPP case was to eat 100% of the time (as in the PP case in predator absence). This removed the potential problem of correlation in the paired action probability parameter sets, as each action probability parameter set evolved to an eat probability of 1. Invasion experiments with these sets of parameters reproduced the same patterns presented in the paper. Thus, both approaches indicate that the higher number of action probability parameters in the PP-consumers relative to the NPP-consumers was inconsequential.
