# Supplementary material for: Phenotypic Plasticity Opposes Species Invasions by Altering Fitness Surface
Source: PLoS Biol. 2006 Oct 31;4(11):e372. doi: 10.1371/journal.pbio.0040372 (PMC1629039; doi:10.1371/journal.pbio.0040372)
Supplement: Supplementary file 5 [file pbio.0040372.sd005.doc]

**Text S5. Invasion Experiments Without Evolution.**

In the invasion experiments described in the main text, initial action probability parameters were random both for the individuals of the resident and invader populations. We chose this method to both allow the species to adapt in a dynamic landscape, and to include evolutionary processes in the invasion dynamics.

We also used an alternative method in order to ensure that the evolutionary aspect of the invasion process was not affecting our results in unforeseen ways; i.e. that our results were robust to the invasion process without evolution. In particular, to investigate the fitness surface mechanism presented in the main text, we determined the optimal behavioral strategy using the genetic algorithm, and then performed the invasion experiments without evolution using species that have a fixed distribution of action probability parameters (without mutations). These invasion experiments were identical to those presented in the text, except all individuals of the resident species had action probability parameters that optimized fitness, whereas the invader species had a distribution of action probability parameters that was a constant distance on the fitness surface away from the optimum. In the PP case, this is all points on a quarter circle equidistant from the peak illustrated in Figure 2a (indicated by a +). In the NPP case, this is two points equidistant, but in opposite directions, from the peak illustrated in Figure 2b and 2d (indicated by a *). When two invading individuals reproduced, the offspring received action probability parameters from these distributions (rather than from parents as previously), and the action probability parameters of the parents were also replaced with new action probability parameters from those distributions to avoid selection in order to keep the distribution unmodified.

This method yielded qualitatively similar results as those presented using the method with evolution as described in the main text. Larger values of the constant distance from the optimal yield longer invasion times, as expected. For a given constant distance, the time for invasion increased for four different combinations of PP-consumers/NPP-consumers and resident/invader pairs (listed Figure 1b) in the same order, and with similar spacing, as that observed with evolution as in the main text. For example, consider an invader species with action probability parameters that are a distance 0.05 from the optimal value, and with a competitive advantage due to the resident having a 20% higher background mortality probability than the invader. In this case, PP-invaders did not invade PP-resident species in any simulations, whereas NPP-invaders invaded a system with NPP-resident rapidly. Indeed, we choose these values as an example because they are similar to those described in the main text. However this finding was general to a large range of the distance from the optimal value, and differences in competitive ability due to different background mortality rates.
